# Fractional Exhaled Nitric Oxide in Children with Non-Cystic Fibrosis Bronchiectasis: Associations with Etiology, Lung Function, and CT Extent

**DOI:** 10.3390/pediatric18030062

**Published:** 2026-05-01

**Authors:** Taner Adiguzel, Bulent Karadag

**Affiliations:** 1 Department of Pediatrics, Faculty of Medicine, Yalova University, Yalova 77200, Türkiye; 2 Department of Pediatric Pulmonology, Faculty of Medicine, Marmara University, Istanbul 34890, Türkiye; bkaradag@marmara.edu.tr

**Keywords:** bronchiectasis, child, exhaled nitric oxide, pediatric pulmonology, primary ciliary dyskinesia, spirometry

## Abstract

Background/Objectives: Childhood non-cystic fibrosis (non-CF) bronchiectasis is clinically heterogeneous. We aimed to describe fractional exhaled nitric oxide (FeNO) levels in affected children and examine associations with etiology, spirometry, and CT-defined disease extent. Methods: This single-center prospective observational study included 100 clinically stable children aged 6–18 years with CT-confirmed non-CF bronchiectasis evaluated between September 2014 and December 2015. FeNO was measured before spirometry using an online single-breath electrochemical technique. Chest CT was reviewed at the lobar level, with the lingula counted separately, and disease extent was summarized by the number of involved lobar regions. Associations were assessed using Spearman correlation and non-parametric tests. Results: Mean age was 14.9 ± 2.0 years, 55% were male, and mean FeNO was 20.9 ± 14.0 ppb. FeNO correlated positively with FEV1 (% predicted), FVC (% predicted), and FEF25–75 (% predicted) (all *p* < 0.01). FeNO was higher in males and adolescents than in females and younger children, respectively. FeNO did not differ by CT-defined lobar extent. It was lower in primary ciliary dyskinesia than in asthma overlap. Overall, 82% of the cohort received an ICS-containing maintenance regimen and household tobacco smoke exposure was present in 58%. Conclusions: FeNO was associated with selected functional indices and etiologic subgroups, but not with CT-defined structural extent, suggesting a greater role in clinical phenotyping than in reflecting radiologic burden. Rather than reflecting overall disease severity, FeNO may be more relevant as a marker of T2-leaning airway inflammatory phenotype in selected children with non-CF bronchiectasis. These findings should be interpreted as exploratory and hypothesis-generating, particularly for etiologic subgroup comparisons and for FeNO interpretation in the setting of treatment and environmental confounding.

## 1. Introduction

Bronchiectasis is a chronic suppurative airway disease characterized by irreversible bronchial dilatation, persistent airway inflammation, and recurrent respiratory morbidity. In children and adolescents, identifying the underlying cause and tracking functional status are central to management and are emphasized in international pediatric guidance [1,2].

In pediatrics, repeated CT imaging is constrained by cumulative radiation exposure, while symptoms and spirometry do not always capture the full biological heterogeneity of disease, making it essential to explore alternative diagnostic methods that can provide a more comprehensive understanding of bronchiectasis in this population. Biomarkers that can be obtained non-invasively, therefore, have practical appeal as adjuncts to phenotyping and longitudinal clinical assessment [3,4,5,6].

Fractional exhaled nitric oxide (FeNO) is widely used in asthma as a marker linked to type 2 airway inflammation and corticosteroid-responsive biology [7,8]. In bronchiectasis, however, the dominant inflammatory pattern is often infection-related and neutrophilic, so FeNO is unlikely to be a direct surrogate of structural severity [9]. At the same time, emerging work suggests that a subgroup of patients with non-CF bronchiectasis may show a type 2 biomarker signal, which may be clinically relevant for phenotyping and targeted clinical evaluation [10,11,12].

Nitric oxide biology is also relevant to primary ciliary dyskinesia (PCD), in which nasal nitric oxide has a well-established role in diagnostic pathways [13,14]. Whether lower-airway nitric oxide measurements provide clinically useful information in children with PCD-related bronchiectasis remains less clear. We therefore aimed to describe FeNO levels in a pediatric non-CF bronchiectasis cohort and to evaluate associations between FeNO and etiologic subgroups, spirometry, and CT-defined extent, summarized by lobar involvement.

## 2. Materials and Methods

### 2.1. Study Design and Setting

This single-center prospective observational study was conducted at the Pediatric Pulmonology outpatient clinic of Marmara University Pendik Training and Research Hospital between September 2014 and December 2015. The study protocol was approved by the Marmara University Faculty of Medicine Research Ethics Committee (approval No. 09.2014.0067; 9 June 2014). Written informed consent for participation was obtained from all participants and/or their parents or legal guardians. The manuscript was prepared in line with the STROBE statement for observational studies [15].

### 2.2. Participants

A total of 100 children aged 6–18 years with CT-confirmed non-cystic fibrosis bronchiectasis who were being followed in the outpatient clinic were included. Eligibility criteria, as specified in the original study protocol, were age 6–18 years, absence of cystic fibrosis, no active respiratory infection within the preceding 3 weeks, and no major coexisting medical diagnosis likely to substantially affect respiratory assessment (e.g., malignancy or cerebral palsy). Participants were evaluated in a clinically stable state and were required to perform FeNO measurement and spirometry reliably. Cystic fibrosis had been excluded by sweat chloride testing and CFTR genetic analysis.

### 2.3. Clinical Data Collection and Etiologic Classification

Demographic and clinical data were collected prospectively using structured study forms at the study visit, and relevant historical and diagnostic information required for etiologic classification was verified from the medical record. Recorded variables included age, sex, age at symptom onset, age at diagnosis, follow-up duration, household tobacco smoke exposure, most recent sputum culture result, bronchiectasis-related surgery history, hospitalizations in the previous year, antibiotic-treated exacerbations in the previous year, maintenance treatment profile, and spirometric measurements. Etiology was classified from the available clinical, laboratory, microbiologic, and imaging data as post-infectious, immunodeficiency, primary ciliary dyskinesia, asthma overlap, idiopathic, or other causes.

### 2.4. Etiologic Work-Up and Microbiology

Etiologic investigation followed the institutional diagnostic approach used in the clinic and was consistent with broad etiologic evaluation recommended in pediatric bronchiectasis care [1]. Depending on clinical indication and data availability, evaluation included sputum or bronchoalveolar lavage microbiology; complete blood count; serum immunoglobulins, including IgG subclasses; HIV testing; assessment for reflux or aspiration; ciliary evaluation from nasal or tracheal epithelium; and bronchoscopy when airway malformation or other endobronchial pathology was suspected. Sputum specimens were collected during routine stable-state outpatient visits before initiation of antibiotic treatment whenever possible, and culture results were categorized descriptively according to growth status or isolated organism.

### 2.5. FeNO Measurement

FeNO was measured before spirometry using a portable electrochemical analyzer (NIOX MINO Airway Inflammation Monitor; Aerocrine AB, Solna, Sweden) with the online single-breath technique at a target expiratory flow of 0.05 L/s, in accordance with ATS/ERS standardized procedures [16]. Measurements were expressed in parts per billion (ppb). Patients were tested in the sitting position. Through the mouthpiece, they inhaled NO-free air for 2–3 s to total lung capacity and then exhaled immediately without breath-holding against the device-generated resistance (>5 cm H_2_O) to maintain a constant expiratory flow and minimize upper-airway contamination. Exhalation time was maintained for at least 6 s to achieve a plateau concentration. Participants were instructed to avoid eating, drinking, and tobacco smoke exposure for at least 1 h before testing.

### 2.6. Spirometry

Spirometry was performed after FeNO measurement using a Winspiro PRO 2.8 system (MIR, Italy, Roma) according to ATS/ERS standardization recommendations [17]. Recorded parameters included forced vital capacity (FVC), forced expiratory volume in 1 s (FEV1), FEV1/FVC ratio, peak expiratory flow (PEF), and forced expiratory flow at 25–75% of FVC (FEF25-75). Percent-predicted values were used for FVC, FEV1, PEF, and FEF25-75 analyses according to the reference equations available in the laboratory software at the time of testing, whereas FEV1/FVC was analyzed as the observed ratio.

### 2.7. Radiological Assessment

Chest CT scans were reviewed at the lobar level, with the lingula considered a separate lobar region. Radiologic extent was summarized according to the number of involved lobar regions as one, two, or more than two involved regions. This pragmatic summary was used for consistency across scans, although dedicated pediatric radiologic scoring systems provide greater granularity [18].

### 2.8. Statistical Analysis

Statistical analyses were performed using SPSS 22. software (IBM Corp., Armonk, NY, USA). Continuous variables were summarized as mean ± standard deviation (SD) and categorical variables as number (percentage). Associations between FeNO and spirometric indices were assessed using Spearman correlation analysis. Because FeNO values were non-normally distributed, group comparisons were performed using the Mann–Whitney U test or Kruskal–Wallis test, as appropriate. Given the exploratory design and limited sizes of some etiologic subgroups, no multivariable modeling or formal adjustment for multiple comparisons was performed. A two-sided *p* value < 0.05 was considered statistically significant. Because no multivariable adjustment was performed and several subgroup comparisons were based on limited sample sizes, all subgroup findings were interpreted as exploratory and hypothesis-generating.

## 3. Results

This study included 100 children and adolescents with non-CF bronchiectasis. The mean age was 14.9 ± 2.0 years, 55% were male, and the mean follow-up duration was 8.0 ± 3.0 years. Symptoms had started at a mean age of 1.75 ± 3.33 years, whereas the mean age at diagnosis was 3.40 ± 1.40 years. Household tobacco smoke exposure was present in 58% of the cohort, and 6% had a history of bronchiectasis-related surgery. During the preceding year, 28% had at least one hospitalization, and 65% had at least one antibiotic-treated exacerbation. Baseline demographic, clinical, FeNO, and spirometric findings are shown in Table 1. Overall, 82% of patients were receiving an ICS-containing maintenance regimen, whereas 18% were not.

The most common etiologic category was post-infectious bronchiectasis (39%), followed by immunodeficiency (23%) and idiopathic disease (19%). Primary ciliary dyskinesia accounted for 9% of cases, asthma overlap for 8%, and other causes for 2%. Sputum culture most commonly showed no bacterial growth (44%) or *Haemophilus influenzae* (32%). Less frequent isolates included *Streptococcus pneumoniae* (11%), *Staphylococcus aureus* (4%), and *Pseudomonas aeruginosa* (4%); other organisms accounted for 5% of cultures. Etiology, sputum microbiology, CT extent categories, and lobar distribution are summarized in Table 2.

On CT, the most frequently involved regions were the left lower lobe (64 patients), right lower lobe (44 patients), and right middle lobe (40 patients). Upper lobe involvement was less frequent, involving the right upper lobe in 30 patients, the lingula in 16 patients, and the left upper lobe in 14 patients. When the radiologic extent was categorized by the number of involved lobar regions, 35 patients had one involved region, 35 had two involved regions, and 30 had more than two involved regions. FeNO levels did not differ significantly across these extent categories (mean values of 19.05, 20.02, and 24.00 ppb, respectively; *p* = 0.510).

FeNO showed significant positive correlations with selected spirometric indices. Higher FeNO values were associated with higher predicted FEV1 (r = 0.371, *p* < 0.001), predicted FVC (r = 0.293, *p* = 0.003), and predicted FEF25-75 (r = 0.271, *p* = 0.006). No significant association was observed between FeNO and FEV1/FVC ratio or PEF in the study dataset. Correlations between FeNO and spirometric indices are summarized in Table 3.

FeNO levels were significantly higher in males than in females (25.34 ± 16.24 vs. 15.42 ± 7.89 ppb, *p* = 0.001). Adolescents aged 12–18 years also had higher FeNO levels than children aged 6 to <12 years (22.32 ± 13.24 vs. 17.81 ± 15.29 ppb, *p* = 0.007). In etiologic subgroup analyses, patients with PCD had lower FeNO levels than those with asthma overlap (13.11 ± 4.91 vs. 26.37 ± 17.88 ppb, *p* = 0.034). Differences between PCD and post-infectious bronchiectasis (13.11 ± 4.91 vs. 23.10 ± 15.46 ppb, *p* = 0.107) and between PCD and non-PCD etiologies overall (13.11 ± 4.91 vs. 21.64 ± 14.39 ppb, *p* = 0.079) did not reach statistical significance. Comparisons of FeNO across key clinical subgroups are presented in Table 4.

## 4. Discussion

In this pediatric non-CF bronchiectasis cohort, mean FeNO was approximately 21 ppb and was associated with selected spirometric indices and etiologic subgroups, whereas no significant association was observed between FeNO and CT extent summarized by involved lobar regions. Taken together, these findings suggest that FeNO may be more informative as a phenotyping adjunct than as a surrogate marker of structural disease burden [9,12].

Interpretation of FeNO in children requires attention to known physiological modifiers. Age- and sex-related variation has been described in healthy pediatric populations, and these factors likely contributed to the higher FeNO values observed in males and in older children in our cohort [19,20]. This point is important when interpreting a single FeNO value across a clinically heterogeneous bronchiectasis population.

The positive associations between FeNO and FEV1, FVC, and FEF25-75 may appear counterintuitive if FeNO is viewed only as a marker of greater inflammation or more severe disease. In asthma, however, FeNO is primarily interpreted as a marker of type 2 airway inflammation and corticosteroid-responsive biology rather than as a direct index of airflow limitation severity [7,8]. Our findings therefore support the possibility that, in a subset of children with bronchiectasis, higher FeNO reflects a distinct inflammatory phenotype rather than greater structural injury. In this context, FeNO should be interpreted primarily as a biomarker of airway inflammatory phenotype, particularly T2-leaning biology, rather than as a general severity marker.

This interpretation is consistent with the increasingly recognized biological heterogeneity of bronchiectasis. Contemporary reviews emphasize that bronchiectasis comprises multiple inflammatory and infective endotypes rather than a single uniform disorder [4,9,21]. Emerging studies have described eosinophilic or type 2 biomarker-positive subgroups in non-CF bronchiectasis and have suggested that FeNO, particularly when interpreted alongside blood eosinophils, may help identify a T2-high endotype [10,11,12]. Our observation that FeNO was higher in the asthma-overlap group than in children with PCD is compatible with this evolving phenotypic model. At the same time, the clinical meaning of a given FeNO value remains context dependent and should be interpreted together with treatment exposure and the broader disease setting.

The lower FeNO levels observed in children with PCD are also biologically plausible. Nitric oxide abnormalities are well recognized in PCD, and nasal nitric oxide has an established role in diagnostic algorithms [13,14]. Although FeNO cannot replace standardized nasal nitric oxide measurement for PCD evaluation, our findings suggest that lower-airway nitric oxide patterns may still provide supportive phenotypic information when interpreted in the appropriate clinical context.

An important finding of the present study is the lack of association between FeNO and CT-defined lobar extent. Structural abnormalities on CT represent cumulative airway damage, whereas FeNO is more likely to reflect contemporaneous airway inflammatory activity. Discordance between these domains is therefore not unexpected in a cross-sectional study design [3,9]. In addition, our radiologic summary was based on the number of involved lobar regions, which is pragmatic but less granular than dedicated pediatric radiologic scoring systems [18].

Our cohort also showed predominantly lower-lobe disease, particularly in the left and right lower lobes, a distribution that is clinically familiar in pediatric non-CF bronchiectasis [1,21]. This anatomic distribution, however, did not translate into a clear FeNO gradient according to radiologic extent in our study, further supporting the concept that FeNO and CT may reflect different disease dimensions.

Another important issue is treatment and environmental confounding. Inhaled corticosteroids may reduce FeNO, whereas poor adherence, concomitant atopy, or ongoing allergic inflammation may increase it despite maintenance therapy [7,8]. In our cohort, 82% of patients were receiving an ICS-containing maintenance regimen and household tobacco smoke exposure was present in 58%. This marked imbalance in ICS exposure, together with heterogeneity in regimen, dose, and duration, limits the interpretability of any unadjusted treatment-based comparison. Parental smoking has also been associated with higher exhaled nitric oxide levels in children [22], and any unadjusted comparison by smoke exposure would risk conflating smoke exposure with age, treatment profile, atopic background, and disease phenotype. Residual confounding is therefore likely, and future prospective studies should incorporate standardized reporting of inhaled corticosteroid exposure, adherence, atopic status, and environmental factors. Because multivariable adjustment was not performed in this study, these associations should be interpreted as exploratory. Systemic corticosteroids may also substantially suppress FeNO, further complicating interpretation when recent rescue therapy has been used [23]. Evidence from biologic-treated airway disease likewise suggests that FeNO behavior is disease- and context-specific rather than uniform across treatment settings [24]. Taken together, these considerations support a cautious, phenotype-oriented interpretation of FeNO as a context-dependent biomarker signal rather than a single disease-severity model. Formal subgroup analyses according to ICS exposure, household tobacco smoke exposure, or individual sputum microbiology categories should therefore be prioritized in future prospective studies.

This study has several limitations. It was conducted at a single center, which may limit generalizability. Some etiologic subgroups, particularly PCD and asthma overlap, were small, reducing statistical power for subgroup comparisons. Although data collection was prospective, the analysis was cross-sectional. We did not have a parallel biomarker panel including blood eosinophils, total IgE, or formal atopy testing, limiting mechanistic interpretation. Finally, CT burden was summarized pragmatically by lobar involvement rather than by a validated quantitative radiologic score. Formal adjusted subgroup analyses according to ICS exposure, household tobacco smoke exposure, or individual sputum microbiology categories were not prespecified in the original statistical plan, and several potentially relevant strata were clinically heterogeneous or small. Recent systemic corticosteroid exposure and other treatment-related modifiers were also not captured in sufficient detail for adjusted analyses. These issues limit causal interpretation and further reinforce the exploratory nature of the findings.

Despite these limitations, the study has practical strengths. It includes a clinically characterized pediatric non-CF bronchiectasis cohort; FeNO was measured under standardized conditions before spirometry, and the analysis addressed functional, etiologic, and radiologic domains within the same dataset. Clinically, FeNO may be most useful not as a severity marker but as a prompt for targeted phenotypic evaluation. Higher FeNO values may encourage closer consideration of asthma overlap or type 2-leaning airway biology, whereas lower values in an appropriate clinical setting may reinforce suspicion for infection-dominant disease or PCD.

## 5. Conclusions

In children with non-CF bronchiectasis, FeNO appears to be more closely related to functional and phenotypic variation, including a possible T2-leaning airway inflammatory signal in selected subgroups, than to CT-defined structural extent. These findings should be considered exploratory. Prospective pediatric studies incorporating standardized CT scoring and multimodal biomarker panels are needed to determine whether FeNO can contribute to clinically actionable phenotyping strategies. Future pediatric studies should incorporate prespecified analyses for corticosteroid exposure, household tobacco smoke exposure, atopic status, blood eosinophils/IgE, and microbiologic status in order to clarify the clinical interpretability of FeNO across biologically and therapeutically heterogeneous subgroups.

## Figures and Tables

**Table 1:** Baseline demographic, clinical, and spirometric characteristics of the stable study cohort.

Variable	Value
Age, years, mean ± SD (range)	14.90 ± 2.02 (7.4–17.8)
Male sex, *n* (%)	55 (55)
Age at symptom onset, years, mean ± SD (range)	1.75 ± 3.33 (0.6–10.0)
Age at diagnosis, years, mean ± SD (range)	3.40 ± 1.40 (1.0–12.7)
Follow-up duration, years, mean ± SD (range)	8.00 ± 3.00 (2–16)
Household tobacco smoke exposure, *n* (%)	58 (58)
Bronchiectasis-related surgery history, *n* (%)	6 (6)
Hospitalizations in the prior year, *n* (%)	0: 72 (72); 1: 14 (14); ≥2: 14 (14)
Antibiotic-treated exacerbations in the prior year, *n* (%)	0: 35 (35); 1–8: 65 (65)
Maintenance therapy, *n* (%)	None: 14 (14); bronchodilator only: 4 (4); bronchodilator + ICS: 58 (58); bronchodilator + ICS + prophylactic antibiotics: 24 (24)
FeNO, ppb, mean ± SD	20.88 ± 14.00
FVC, % predicted, mean ± SD	72.76 ± 21.43
FEV1, % predicted, mean ± SD	70.74 ± 23.66
FEV1/FVC, %, mean ± SD	97.32 ± 12.55
PEF, % predicted, mean ± SD	69.82 ± 27.65
FEF25-75, % predicted, mean ± SD	73.58 ± 36.35

Participants are assessed in a clinically stable state; active respiratory infection within the preceding 3 weeks is an exclusion criterion. Spirometric values are presented as percent predicted, except FEV1/FVC, which is shown as the observed ratio. Data are presented as mean ± SD (range) or *n* (%). FeNO, fractional exhaled nitric oxide; FEF25-75, forced expiratory flow at 25–75% of forced vital capacity; FEV1, forced expiratory volume in 1 s; FVC, forced vital capacity; ICS, inhaled corticosteroid; PEF, peak expiratory flow.

**Table 2:** Etiology, sputum microbiology, and CT findings.

Domain	Category	Value
Etiology	Post-infectious	39 (39)
Etiology	Immunodeficiency	23 (23)
Etiology	Idiopathic	19 (19)
Etiology	Primary ciliary dyskinesia	9 (9)
Etiology	Asthma overlap	8 (8)
Etiology	Other causes	2 (2)
Sputum culture	No growth	44 (44)
Sputum culture	*Haemophilus influenzae*	32 (32)
Sputum culture	*Streptococcus pneumoniae*	11 (11)
Sputum culture	*Staphylococcus aureus*	4 (4)
Sputum culture	*Pseudomonas aeruginosa*	4 (4)
Sputum culture	Other	5 (5)
CT extent	One involved lobar region	35 (35)
CT extent	Two involved lobar regions	35 (35)
CT extent	>2 involved lobar regions	30 (30)
CT distribution	Left lower lobe	64
CT distribution	Right lower lobe	44
CT distribution	Right middle lobe	40
CT distribution	Right upper lobe	30
CT distribution	Lingula	16
CT distribution	Left upper lobe	14

CT distribution values indicate the number of patients with involvement in each lobar region; totals exceed 100 because more than one lobar region could be involved in an individual patient.

**Table 3:** Correlations between FeNO and spirometric indices.

Analysis	Group/Variable	Result	*p* Value
Correlation (Spearman)	FEV1 (% predicted)	r = 0.371	<0.001
Correlation (Spearman)	FVC (% predicted)	r = 0.293	0.003
Correlation (Spearman)	FEF25-75 (% predicted)	r = 0.271	0.006

Only statistically significant correlations are shown. FeNO, fractional exhaled nitric oxide; FEF25-75, forced expiratory flow at 25–75% of forced vital capacity; FEV1, forced expiratory volume in 1 s; FVC, forced vital capacity.

**Table 4:** FeNO across clinical subgroups.

Analysis	Group/Variable	Result	*p* Value
Sex comparison	Male vs. female	25.34 ± 16.24 vs. 15.42 ± 7.89 ppb	0.001
Age group comparison	12–18 years vs. 6 to <12 years	22.32 ± 13.24 vs. 17.81 ± 15.29 ppb	0.007
CT extent	One (*n* = 35) vs. two (*n* = 35) vs. >2 (*n* = 30)	Mean FeNO: 19.05 vs. 20.02 vs. 24.00 ppb	0.510
Etiology comparison	PCD vs. asthma overlap	13.11 ± 4.91 vs. 26.37 ± 17.88 ppb	0.034
Etiology comparison	PCD vs. post-infectious	13.11 ± 4.91 vs. 23.10 ± 15.46 ppb	0.107
Etiology comparison	PCD vs. non-PCD etiologies	13.11 ± 4.91 vs. 21.64 ± 14.39 ppb	0.079

Non-parametric tests are used for group comparisons because FeNO distributions are non-normal. Group comparison results are shown as mean ± SD to preserve consistency with the original dataset summary. Because no multivariable adjustment is performed, pairwise etiologic comparisons should be interpreted as exploratory. CT, computed tomography; PCD, primary ciliary dyskinesia.

## Data Availability

The data presented in this study are available upon request from the corresponding author. The data are not publicly available because they derive from pediatric patient records and contain potentially identifiable clinical information.

## Abbreviations

CFTR, cystic fibrosis transmembrane conductance regulator; CT, computed tomography; FeNO, fractional exhaled nitric oxide; FEF25-75, forced expiratory flow at 25–75% of forced vital capacity; FEV1, forced expiratory volume in 1 s; FVC, forced vital capacity; HIV, human immunodeficiency virus; ICS, inhaled corticosteroid; PCD, primary ciliary dyskinesia; PEF, peak expiratory flow; ppb, parts per billion; SD, standard deviation; and STROBE, Strengthening the Reporting of Observational Studies in Epidemiology.
